# Weak cough is associated with increased mortality in COPD patients with scheduled extubation: a two-year follow-up study

**DOI:** 10.1186/s12931-022-02084-9

**Published:** 2022-06-23

**Authors:** Yueling Hong, Min Deng, Wenhui Hu, Rui Zhang, Lei Jiang, Linfu Bai, Jun Duan

**Affiliations:** Department of Respiratory and Critical Care Medicine, The First Affiliated Hospital of Chongqing Medical University, Youyi Road 1, Yuzhong District, Chongqing, 400016 People’s Republic of China

**Keywords:** Cough strength, Mechanical ventilation, Mortality

## Abstract

**Background:**

Cough strength is associated with short-term outcome in patients with scheduled extubation who successfully complete a spontaneous breathing trial (SBT). However, the long-term outcome is unclear.

**Methods:**

This was a prospective observational study performed in a respiratory ICU of a teaching hospital. COPD patients who successfully completed a SBT were candidates. We enrolled the case who assessed the cough strength by cough peak flow (CPF) or semiquantitative cough strength score (SCSS, ranging from 0 = weak to 5 = strong). Patients were followed up to two years by phone every 3 months.

**Results:**

A total of 215 patients were enrolled in current study. Among them, CPF and SCSS were measured in 214 and 208 cases, respectively. Strong cough was associated with a 16% decrease in the risk of two-year mortality (adjusted hazard ratio [HR] 0.84, 95%CI: 0.78–0.91) per 10 L/min increment of CPF. When it was tested by SCSS, decrease in the risk of two-year mortality per unit increment was 27% (adjusted HR 0.73, 95%CI: 0.62–0.86). Similar results were confirmed in the discharged patients. In all patients, the two-year mortality was 75%, 53%, and 38% in patients with CPF < 60, 60–90, and > 90 L/min; and 85%, 70%, and 40% in patients with SCSS of 0–1, 2–3, and 4–5, respectively. Similar trend was found among the discharged patients whether it was assessed by CPF or SCSS.

**Conclusions:**

In COPD patients, weak cough is associated with increased two-year mortality after a scheduled extubation. It provides objective information to caregivers to improve decision-making process during hospitalization and after discharge.

## Background

Endotracheal intubation is the main interface for invasive mechanical ventilation. When the respiratory failure is reversed, weaning from mechanical ventilation is considered. Spontaneous breathing trial (SBT) has been recommended to access whether the patient has the ability to maintain spontaneous breath without ventilator support [1–3]. After a successful SBT, endotracheal intubation has been removed. Many studies have reported the short-term outcome among patients with a successful SBT [4–7]. However, few studies focused on the long-term outcome.

Weak cough is a strong predictor of extubation failure in patients with a successful SBT [8]. It can be measured by cough peak flow (CPF) when the staff coaches the patient to cough through the endotracheal tube as strong as possible [9]. It also can be measured by a semiquantitative cough strength scale (SCSS) [10]. However, the long-term outcome is unclear among the patients with weak cough. Here, we aimed to explore the risk of death within two years after a successful SBT in COPD patients with various cough strength assessed by CPF and SCSS.

## Methods

This was a prospective observational study performed in a respiratory intensive care unit (ICU) of a teaching hospital. Patients with acute exacerbation of chronic obstructive pulmonary disease (COPD) were candidates. We enrolled those who completed a successful SBT and was ready for extubation. However, patients who had a tracheotomy or were lost to follow up were also excluded. The study protocol was approved by our local ethics committee and institutional review board (approval date: May 13th, 2011 and November 10th, 2016). As the observational nature, informed consent was waived.

Patients were managed according to current guidelines and our hospital’s protocols [2, 3, 9, 11–13]. We used propofol, midazolam, dexmedetomidine, fentanyl and/or morphine to manage sedation and analgesia. The target was to maintain a Ramsay score of 3 to 4 or a Richmond agitation sedation scale of − 2 to 0. Strategies such as early exercise and mobilization to speed extubation, subglottic suctioning, elevation of the head of the bed, and hand hygiene were used to prevent ventilator-associated pneumonia. Tube feeding was given according to the energy consumption. Fluid resuscitation was given to hypotensive patients. And fluid limitation was given to the patients with excessive extravascular lung water.

The respiratory therapists and physicians screened the patients every morning to identify the candidates who would initiate the weaning process. The criteria for initiation of weaning process were as follows: improvement in the underlying condition that led to acute respiratory failure; PaO_2_/FiO_2_ > 150 mm Hg in patients with chronic hypoxia or > 200 mm Hg in those with acute hypoxia; requirement of FiO_2_ ≤ 0.5; pH ≥ 7.35; positive-end expiratory pressure (PEEP) < 8 cmH_2_O; temperature < 38 °C; systolic blood pressure between 90 and 180 mm Hg (without vasopressor therapy or with only a low-dose vasopressor such as dopamine or dobutamine < 5 ug/kg/min); heart rate < 140 beats/min; and breathing frequency < 30 breaths/min.

If the patients reached the criteria for initiation of the weaning process, a SBT was performed for 30 to 120 min. The method of SBT was low level of pressure support ventilation (6–8 cm H_2_O). If the patient experienced an unsuccessful SBT, the pervious ventilation parameters were used and weaning attempt was assessed next day. The criteria for an unsuccessful SBT were as follows: respiratory rate > 35 breaths/min; rapid shallow breathing index (f/Vt) > 105; SpO_2_ < 90% with a FiO_2_ > 0.5; heart rate > 140 or < 50 beats/min; systolic blood pressure > 180 or < 90 mm Hg; pH < 7.3; diminishing consciousness or diaphoresis; and clinical signs indicating respiratory muscle fatigue, labored breathing, or both.

Extubation was performed when the patient successfully completed a SBT. Before extubation, cough strength was assessed. Before measurement, the head of the bed was elevated at 30–45°, and secretions were removed by suction. An external flowmeter (Chestgraph HI-101, Chest MI, Tokyo, Japan) or a built-in ventilator flowmeter (PB840, Covidien, Mansfield, Massachusetts) were used to measure CPF [9]. For external flowmeter, we disconnected the ventilator, connected the flowmeter to the endotracheal tube, and coached the patient to cough with as much effort as possible. For built-in ventilator flowmeter, the parameters of the ventilator were the same as those in a SBT. We also coached the patient to cough with as much effort as possible. At the same time, we froze the waveform of the flow velocity. Then we visually picked the peak of the flow velocity from the graph and kept the number to single digits. The best of 3 values was recorded.

To measure SCSS, we coached the patient to cough with as much effort as possible when the ventilator was disconnected. The SCSS ranged from 0 to 5: 0 = no cough on command, 1 = audible movement of air through the endotracheal tube but no audible cough, 2 = weakly (barely) audible cough, 3 = clearly audible cough, 4 = stronger cough and 5 = multiple sequential strong coughs [10]. After extubation, prophylactic use of noninvasive ventilation (NIV) or high flow nasal cannula (HFNC) was performed in patients at high risk for extubation failure [14–16]. All the patients with a scheduled extubation were followed up every 3 months to two years after extubation.

### Statistical analysis

Patients were classified to low, moderate and strong cough, respectively, if the CPF < 60, 60–90 and > 90 L/min, or SCSS of 0–1, 2–3 and 4–5 [17, 18]. Qualitative and categorical variables were reported as numbers and percentages, and differences between groups were compared with the χ^2^ test or Fisher’s exact test, as appropriate. Continuous variables were reported as mean values and standard deviations or median values and interquartile ranges, as appropriate. Differences between groups were compared with one-way ANOVA or the Kruskal–Wallis H test. Cox regression was used to analyze the risk for two-year mortality. Directed acyclic graphs (DAGs) were used to select variables that were introduced in the multivariable model as potential confounders [19, 20]. Browser-based software, DAGitty (http://www.dagitty.net), was used to create DAGs. The cumulative two-year survival probability was analyzed by creating Kaplan–Meier curves. The results were presented as hazard ratios (HR) with 95% confidence interval (CI). Statistical significance was defined as P ≤ 0.05.

## Results

**Fig. 1:**
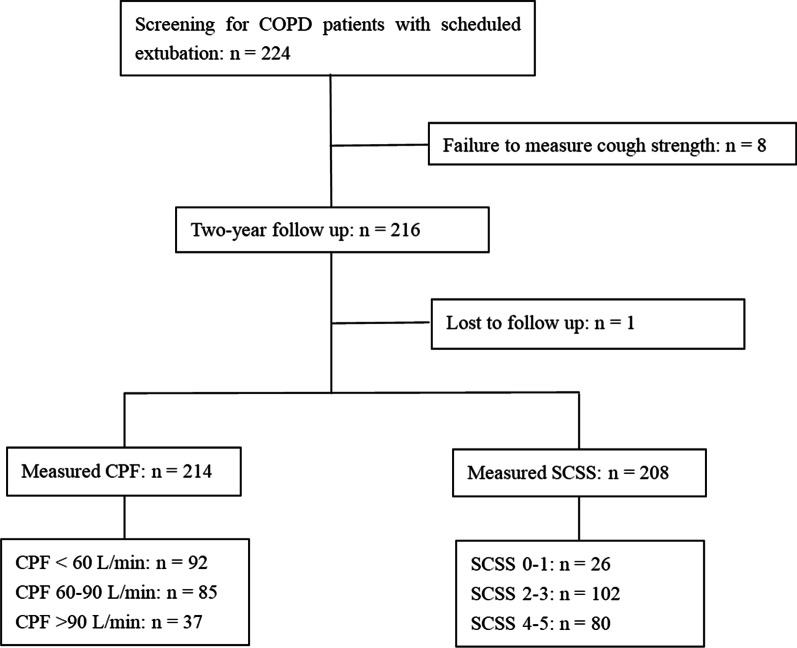
Flow of patient screening and enrollment. *CPF*  cough peak flow, *SCSS*  semiquantitative cough strength score

From February 2011 to November 2019, we screened 224 COPD patients with scheduled extubation (Fig. 1). However, we excluded 8 patients due to failure to measure cough strength and one patient due to lost during two-year follow up. Therefore, 215 patients were enrolled in current study. Among them, CPF were measured in 214 cases (212 for external flowmeter and 2 for built-in ventilator flowmeter). SCSS was measured in 208 cases. There were 92 (43%), 85 (40%) and 37 (17%) patients with CPF < 60, 60–90 and > 90 L/min, respectively. Among patients who measured SCSS, 26 (13%), 102 (49%), 80 (38%) cases had SCSS of 0–1, 2–3, and 4–5, respectively.

Patients with weak cough were older than those with strong cough (Table 1). They also had higher Charlson comorbidity index, higher proportion of difficult and prolonged weaning, longer duration of mechanical ventilation before extubation, higher proportion of reintubation at 72 h after extubation, and higher duration of hospital stay after extubation than the cases with strong cough.

**Table 1:** Baseline data

	CPF (L/min), N = 214			*p*	SCSS, N = 208			*p*
	< 60 N = 92	N = 85	> 90 N = 37		0–1 N = 26	2–3 N = 102	4–5 N = 80	
Age, years	76 ± 8	72 ± 9	68 ± 11	< 0.01	81 ± 7	74 ± 8	69 ± 10	< 0.01
Male	64 (70%)	73 (86%)	35 (95%)	< 0.01	19 (73%)	78 (77%)	70 (88%)	0.11
Charlson comorbidity index	2 (1–3)	1 (1–2)	1 (0–2)	0.02	2 (1–3)	2 (1–2)	1 (1–2)	< 0.01
Weaning category								
Simple weaning	47 (51%)	62 (73%)	28 (76%)	0.02	11 (42%)	61 (60%)	59 (74%)	< 0.01
Difficult weaning	37 (40%)	20 (24%)	8 (22%)		10 (39%)	37 (36%)	18 (23%)	
Prolonged weaning	8 (9%)	3 (4%)	1 (3%)		5 (19%)	4 (4%)	3 (4%)	
Variables collected before extubation								
Duration of MV before extubation, d	6 (4–9)	5 (3–6)	5 (3–7)	0.04	8 (6–10)	5 (3–8)	4 (3–6)	< 0.01
PEEP, cmH_2_O	5 (4–6)	5 (4–6)	5 (4–5)	0.44	5 (5–6)	5 (4–6)	5 (4–5)	0.10
APACHE II score	13 ± 3	12 ± 3	11 ± 3	< 0.01	14 ± 3	13 ± 3	11 ± 3	< 0.01
Hemoglobin, g/dl	11.1 ± 2.2	12.0 ± 2.1	12.8 ± 2.0	< 0.01	10.2 ± 2.1	11.6 ± 2.2	12.5 ± 2.1	< 0.01
Albumin, g/L	30 ± 4	31 ± 5	32 ± 5	0.04	30 ± 4	30 ± 4	31 ± 5	0.44
Respiratory rate, breaths/min	22 ± 5	21 ± 5	22 ± 6	0.67	23 ± 5	21 ± 5	21 ± 5	0.35
Rapid shallow breathing index	59 ± 25	51 ± 24	50 ± 24	0.06	61 ± 23	58 ± 26	49 ± 23	0.02
Heart rate, beats/min	96 ± 16	99 ± 16	95 ± 13	0.23	96 ± 17	99 ± 15	96 ± 16	0.37
Systolic blood pressure, mmHg	134 ± 22	135 ± 22	132 ± 22	0.74	133 ± 17	136 ± 24	132 ± 21	0.47
Diastolic blood pressure, mmHg	70 ± 12	74 ± 12	73 ± 12	0.03	67 ± 10	73 ± 12	74 ± 12	0.03
PH	7.42 ± 0.05	7.41 ± 0.05	7.42 ± 0.05	0.47	7.43 ± 0.06	7.41 ± 0.05	7.42 ± 0.05	0.41
PaCO_2,_ mmHg	54 ± 11	56 ± 11	51 ± 12	0.12	50 ± 12	56 ± 11	53 ± 12	0.02
PaO_2_/FiO_2,_ mmHg	232 ± 71	212 ± 55	212 ± 48	0.06	249 ± 67	223 ± 67	210 ± 50	0.02
Prophylactic use of NIV or HFNC	68 (74%)	70 (82%)	20 (54%)	< 0.01	17 (65%)	82 (80%)	55 (69%)	0.12
Reintubation at 72 h	14 (15%)	2 (2%)	2 (5%)	< 0.01	7 (27%)	6 (6%)	4 (5%)	< 0.01
Duration of hospital stay, days	21 (14–31)	17 (12–26)	17 (11 -22)	0.04	21 (15–25)	19 (13–28)	17 (10–23)	0.03
Duration of ICU stay, days	14 (10–23)	12 (7–16)	9 (6–12)	< 0.01	14 (12–23)	13 (8–22)	9 (6–15)	< 0.01
Duration of hospital stay after extubation, days	12 (7–18)	11 (6–17)	10 (6–13)	0.21	9 (5–14)	11 (8–19)	10 (5–16)	0.08
Duration of ICU stay after extubation, days	7 (4–14)	6 (3–9)	4 (2–8)	< 0.01	7 (4–11)	7 (5–11)	5 (2–8)	< 0.01

*CPF*  cough peak flow, *SCSS*  semiquantitative cough strength score, *MV*  mechanical ventilation, *NIV*  noninvasive ventilation, *HFNC*  high-flow nasal cannula, *ICU*  intensive care unit, *PEEP*  positive-end expiratory pressure

Two-year mortality was 59.8% and 60.1% in scheduled extubation patients who measured CPF and SCSS, respectively. In patients with CPF < 60, 60–90, and > 90 L/min, it was 75%, 53% and 38%, respectively (Table 2). Among the patients who measured SCSS, two-year mortality was 85%, 70%, and 40% in the cases with SCSS of 0–1, 2–3, and 4–5, respectively. There were 173 discharged patients tested CPF and two-year mortality was 50.2%. For the 167 discharged patients measured SCSS, two-year mortality was 50.3%. The two-year mortality was 62%, 49%, and 32% in discharged patients with CPF < 60, 60–90, and > 90 L/min, and 67%, 62%, and 34% in discharged patients with SCSS of 0–1, 2–3 and 4–5, respectively.

**Table 2:** Mortality within two years after extubation

	CPF (L/min), N = 214			*p*	SCSS, N = 208			*p*
Mortality in all patients	< 60 N = 92	60–90 N = 85	> 90 N = 37		0–1 N = 26	2–3 N = 102	4–5 N = 80	
One Month	28 (30%)	8 (9%)	2 (5%)	< 0.01	13 (50%)	16 (16%)	8 (10%)	< 0.01
Three months	40 (44%)	13 (15%)	2 (5%)	< 0.01	17 (65%)	28 (28%)	9 (11%)	< 0.01
Six months	45 (49%)	23 (27%)	5 (14%)	< 0.01	19 (73%)	35 (34%)	18 (23%)	< 0.01
One year	54 (59%)	34 (40%)	8 (22%)	< 0.01	21 (81%)	49 (48%)	24 (30%)	< 0.01
Two years	69 (75%)	45 (53%)	14 (38%)	< 0.01	22 (85%)	71 (70%)	32 (40%)	< 0.01

	CPF (L/min), N = 173			*p*	SCSS, N = 167			*p*
Mortality in discharged patients	< 60 N = 60	60–90 N = 79	> 90 N = 34		0–1 N = 12	2–3 N = 82	4–5 N = 73	
One Month	3 (5%)	3 (4%)	0 (0%)	0.43	1 (8%)	2 (2%)	2 (3%)	0.53
Three months	9 (15%)	7 (9%)	0 (0%)	0.05	4 (33%)	8 (10%)	3 (4%)	< 0.01
Six months	14 (23%)	17 (22%)	3 (9%)	0.20	6 (50%)	15 (18%)	12 (16%)	0.02
One year	22 (37%)	28 (35%)	5 (15%)	0.06	7 (58%)	29 (35%)	17 (23%)	0.03
Two years	37 (62%)	39 (49%)	11 (32%)	0.02	8 (67%)	51 (62%)	25 (34%)	< 0.01

*CPF* cough peak flow, *SCSS* semiquantitative cough strength score

**Fig. 2:**
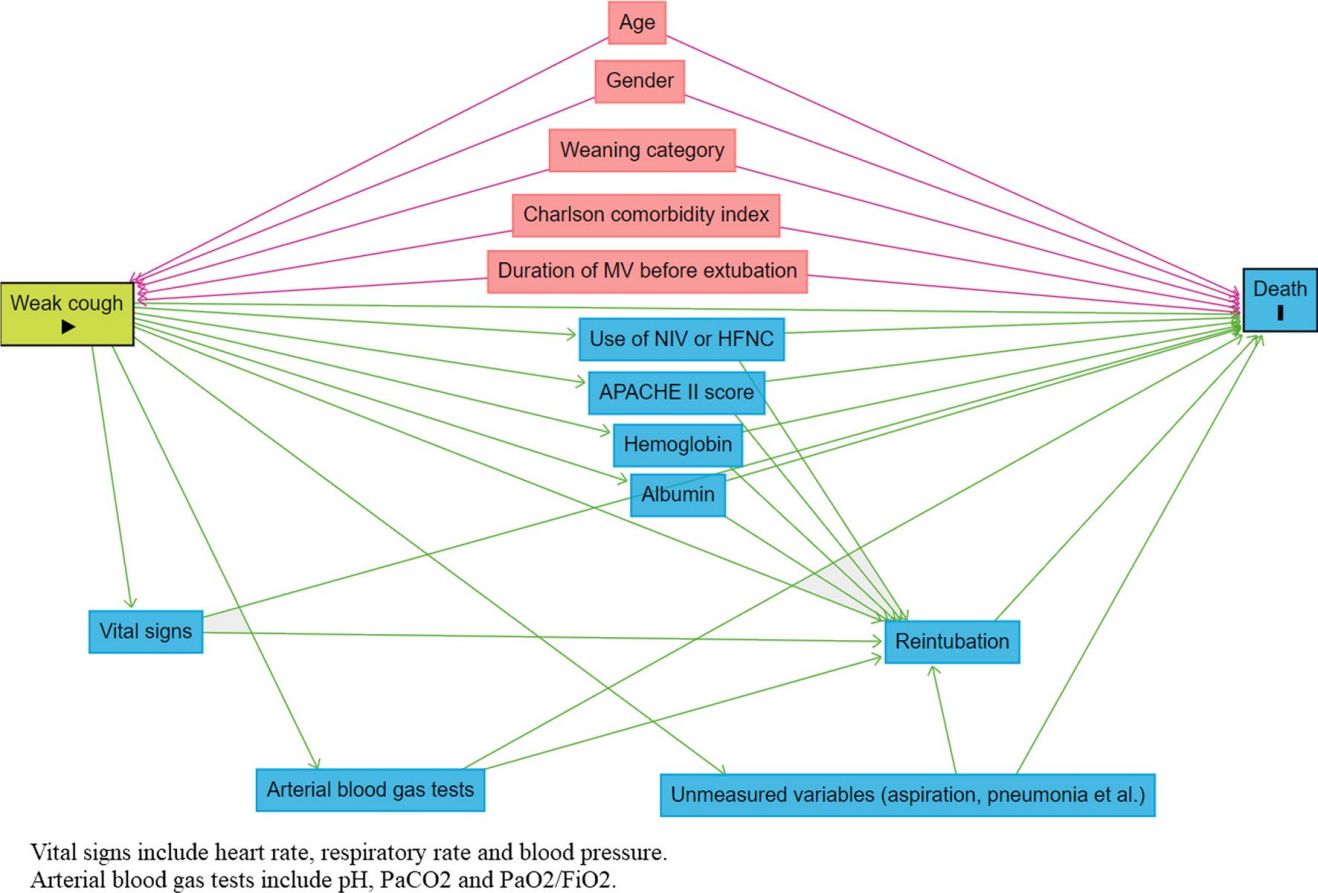
Confounders were identified by the directed acyclic graphs to explore the association between weak cough and death. The red nodes above represent confounders and have been used to adjust the hazard ratio between weak cough and death. The blue nodes below represent mediators and cannot be used to adjust the hazard ratio. *MV*  mechanical ventilation, *NIV*  noninvasive ventilation, *HFNC*  high-flow nasal cannula

**Fig. 3:**
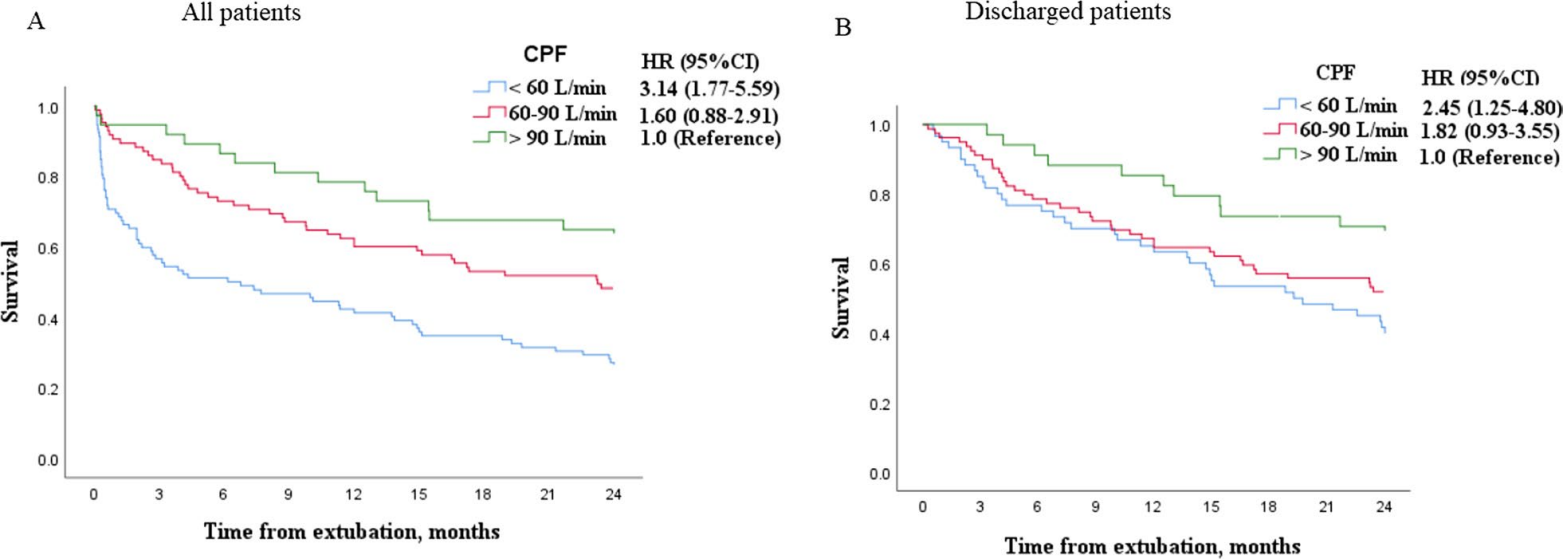
Two-year survival in patients with cough peak flow (CPF) < 60, 60–90 and > 90 L/min. *HR*  hazard ratio, *CI*  confidence interval

Confounders were identified by DAGs to explore the association between weak cough and two-year mortality (Fig. 2). In the overall cohort, the crude HR of two-year mortality was 0.81 (95%CI: 0.76–0.87) per 10 L/min increment of CPF. When it was adjusted by confounders, the HR was 0.84 (95%CI: 0.78–0.91) (Table 3). Compared with patients with CPF > 90 L/min, the HR of two-year mortality was 1.60 (95%CI: 0.88–2.91) and 3.14 (1.77–5.59) in patients with CPF 60–90 and > 90 L/min, respectively (Fig. 3). Similar results were found in the discharged patients.

**Table 3:** Cox regression analysis for two-year mortality

	Overall cohort		Discharged patients	
	Crude HR (95%CI)	Adjusted HR (95%CI)^a^	Crude HR (95%CI)	Adjusted HR (95%CI)^a^
CPF, per 10 L/min increase	0.81 (0.76–0.87)	0.84 (0.78–0.91)	0.86 (0.79–0.93)	0.89 (0.81–0.97)
SCSS, per unit increase	0.68 (0.59–0.78)	0.73 (0.62–0.86)	0.72 (0.60–0.86)	0.81 (0.66–0.99)

*HR*  hazard ratio, *CI*  confidence interval, *CPF*  cough peak flow, *SCSS*  semiquantitative cough strength score

^a^HR was adjusted by age, sex, Charlson comorbidity index, weaning category and duration of mechanical ventilation before extubation

**Fig. 4:**
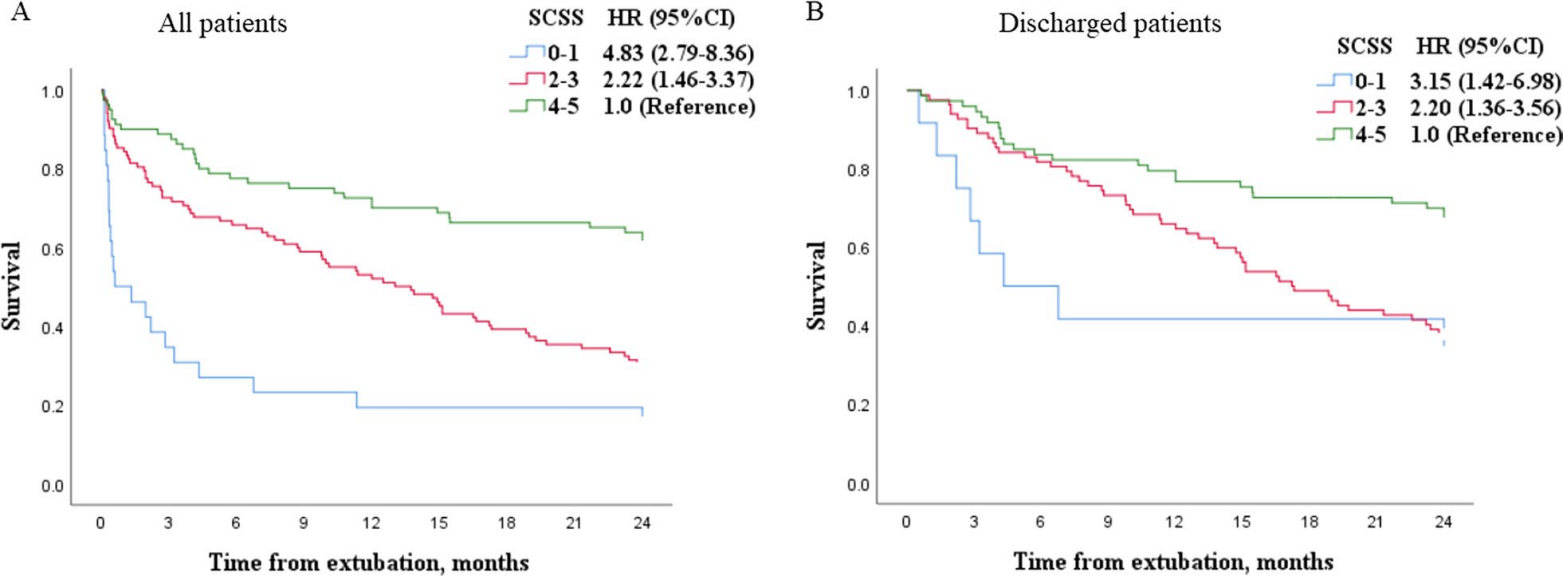
Two-year survival in patients with semiquantitative cough strength score (SCSS) of 0–1, 2–3 and 4–5. *HR*  hazard ratio, *CI*  confidence interval

Among the patients who measured SCSS, the crude and adjusted HR of two-year mortality was 0.68 (95%CI: 0.59–0.78) and 0.73 (0.62–0.86) per unit increment, respectively. Compared with patients with SCSS of 4–5, the HR of 2 year mortality was 2.22 (95%CI: 1.46–3.37) and 4.83 (2.79–8.36) in patients with SCSS 2–3 and 0–1, respectively (Fig. 4). The results were confirmed in the discharged patients.

## Discussion

Current study shows that 60% of COPD patients with a scheduled extubation after a successful SBT died within two years after extubation. Cough strength measured by CPF or SCSS is strongly associated with two-year mortality. Weaker cough indicates higher risk for two-year mortality.

CPF is most commonly used and SCSS is secondary used to measure cough strength for prediction of extubation failure [8]. Weaker cough is associated with higher risk of extubation failure. In our study, we confirmed the results that patients with weaker cough had higher risk of reintubation. For the high risk patients, preventive use of NIV or HFNC is an alternative strategy to avoid reintubation [14–16].

Two-year mortality is 51% in general ICU survivors ≥ 65 years who received mechanical ventilation [21]. In our study, the two-year mortality was 60% in COPD patients with scheduled extubation and 50% in the discharged ones. Due to chronic lung disease, the two-year mortality was higher than the previous study [21]. In addition, increased age, prolonged mechanical ventilation and co-morbidities are associated with long-term outcomes [22–24]. We counted these factors as confounders. After adjustment of these confounders, we still found weak cough was strongly associated with two-year mortality. Therefore, the assessment of cough strength is important. For patients with weak cough, more attention should be paid such as aspiration. For the studies focused on prognosis, cough strength should be considered as a confounder to adjust the risk of interesting events (e.g. reintubation and mortality).

Cough strength is positively correlated with maximal inspiratory pressure (MIP) [25]. One-year mortality is 31% in scheduled extubation patients with low MIP (< 30 cmH_2_O) and 7% in those with high MIP (≥ 30 cmH_2_O) [26]. This is one reason for higher two-year mortality in patients with lower cough strength. In addition, weak cough also increases the risk of pneumonia within one year and aspiration is the most probable reason [27–29]. Moreover, persistent sputum production is a common feature in COPD patients [30, 31]. Weak cough diminishes the ability of airway clearance, which increases the risk for plugging, atelectasis and apnea. These reasons can further explain the association between weak cough and increased two-year mortality.

Airway clearance technologies such as positive expiratory pressure (PEP) devices or directed huffing increase sputum expectoration [32]. This can reduce the risk for sputum plugging and pulmonary atelectasis. In addition, pulmonary rehabilitation and inspiratory muscle training can improve the MIP [33, 34]. Higher MIP indicates higher cough strength. Therefore, airway clearance technologies, pulmonary rehabilitation and inspiratory muscle training may benefit patients with weak cough.

Current study had several limitations. First, only COPD patients were enrolled in current study. The results cannot be extrapolated to other patients. Second, we only reported the association between cough strength and two-year mortality. The quality of life is unclear. It is encouraged to explore the association between cough strength and quality of life. Third, the weight, body mass index, delirium and fluid balance may influence the measurement of cough strength and extubation. Failure to collect these variables may skew the results. Fourth, we did not record the cause of death. It is unable to determine the mechanism of weak cough and death. Further study should explore the mechanism why weak cough increases two-year mortality. Fifth, the BODE index or ADO index, which was assessed in non-critically ill patients with COPD, was associated with poor prognosis [35, 36]. Current study was focused on critically ill patients with COPD. In ICUs, it was unable to measure pulmonary function test or 6-min walking test. So, we did not collect BODE index or ADO index. However, we have collected Charlson Comorbidity index and age as confounders, which can partly reflect the originally frail.

## Conclusion

Sixty percent of COPD patients with a scheduled extubation after a successful SBT died within two years after extubation. Among the discharged patients, two-year mortality was still as high as 50%. Weak cough tested by CPF or SCSS was associated with increased two-year mortality.

## Data Availability

The datasets analyzed during the current study available from the corresponding author on reasonable request.

## Abbreviations

SBT: Spontaneous breathing trial
CPF: Cough peak flow
SCSS: Semiquantitative cough strength score
PEEP: Positive-end expiratory pressure
AECOPD: Acute exacerbation of chronic obstructive pulmonary disease
MV: Mechanical ventilation
NIV: Noninvasive ventilation
HFNC: High-flow nasal cannula
ICU: Intensive care unit
HR: Hazard ratio
CI: Confidence interval
